# Protocol for generating airway organoids from 2D air liquid interface-differentiated nasal epithelia for use in a functional CFTR assay

**DOI:** 10.1016/j.xpro.2023.102337

**Published:** 2023-06-12

**Authors:** Lisa W. Rodenburg, Isabelle S. van der Windt, Henriette H.M. Dreyer, Shannon M.A. Smits, Loes A. den Hertog - Oosterhoff, Ellen M. Aarts, Jeffrey M. Beekman, Gimano D. Amatngalim

**Affiliations:** 1 Department of Pediatric Pulmonology, Wilhelmina Children’s Hospital, University Medical Center Utrecht, Utrecht University, Member of ERN-LUNG, Utrecht EA 3584, the Netherlands; 2 Regenerative Medicine Center Utrecht, University Medical Center Utrecht, Utrecht University, Utrecht CB 3584, the Netherlands; 3 Centre for Living Technologies, Alliance TU/e, WUR, UU, UMC Utrecht, Utrecht CB 3584, the Netherlands

**Keywords:** Cell culture, Organoids

## Abstract

We present a protocol to generate organoids from air-liquid-interface (ALI)-differentiated nasal epithelia. We detail their application as cystic fibrosis (CF) disease model in the cystic fibrosis transmembrane conductance regulator (CFTR)-dependent forskolin-induced swelling (FIS) assay. We describe steps for isolation, expansion and cryostorage of nasal brushing-derived basal progenitor cells, and their differentiation in ALI cultures. Furthermore, we detail the conversion of differentiated epithelial fragments into organoids of healthy controls and CF subjects for validating CFTR function and modulator responses.

For complete details on the use and execution of this protocol, please refer to Amatngalim et al.^1^

## Highlights

• Procedures for culturing nasal brushing-derived basal progenitor cells
• Technique to convert 2D ALI-differentiated nasal epithelia into organoids
• Prediction of CFTR modulator drug efficacy in CF nasal organoids in FIS assays

## Abstract (editor highlights)

Publisher’s note: Undertaking any experimental protocol requires adherence to local institutional guidelines for laboratory safety and ethics.

## Graphical abstract

**Figure:**
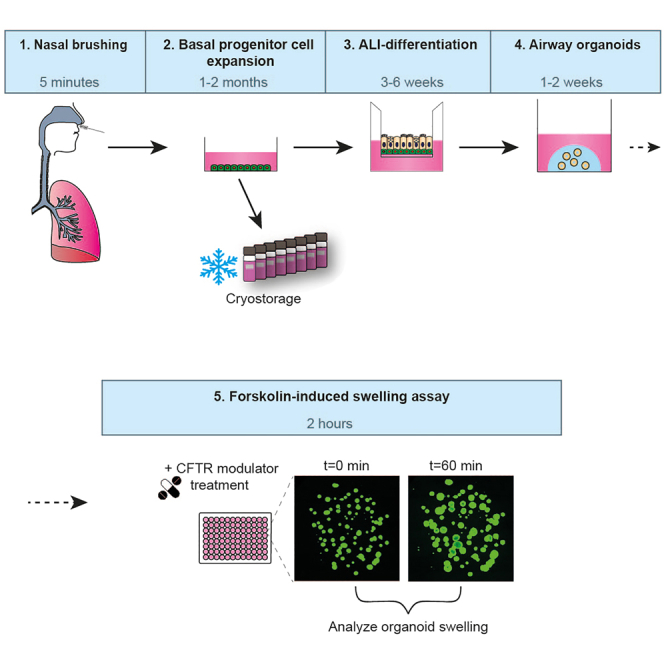

## Before you begin

### Institutional permissions

All human samples used for this study were acquired with informed consent of the subjects and approval of a specific ethical board for the use of biobanked materials TcBIO (Toetsingscommissie Biobanks), an institutional Medical Research Ethics Committee of the University Medical Center Utrecht (protocol ID: 16/586).

**CRITICAL:** This protocol describes the use of primary human nasal epithelial cells. It is important that permission has been received from relevant institutions and it should meet regulatory standards, including informed consent from all subjects.

### Preparation: Precoating of well plates for basal progenitor cell expansion

**Timing: 1.5–24 h**

1. Coat wells in a 12- or 6-well plate with collagen IV (50 μg/mL in PBS0) and incubate at 37°C and 5% CO_2_ for 1–24 h.
  a. For one well in a 12-well plate: add 500 μL collagen IV solution.
  b. For one well in a 6-well plate: add 1000 μL collagen IV solution.

***Note:*** After 24 h of incubation with the coating, precoated wells can be stored in the fridge for a maximum of 14 days. For storage, replace the collagen coating solution with 1 mL (12-well plate) or 2 mL (6-well plate) PBS0, wrap the plate in foil and store at 4°C.

### Preparation: Precoating of transwell inserts for ALI-differentiation

**Timing: 1.5–24 h**

2. Coat Transwell inserts at the apical side with PureCol (30 μg/mL in PBS0) and incubate at 37°C and 5% CO_2_ for 1–24 h.
  a. For one 6.5 mm insert (24-well plate): add 200 μL PureCol solution.
  b. For one 12 mm insert (12-well plate): add 400 μL PureCol solution.

***Note:*** After 24 h of incubation with the coating, precoated wells can be stored in the fridge for a maximum of 14 days. For storage, replace the PureCol coating solution with 300 μL (6.5 mm insert) or 600 μL (12 mm insert) PBS0, fill the basolateral compartment with PBS0, wrap the plate in foil and store at 4°C.

## Key resources table

**Table:** 

REAGENT or RESOURCE	SOURCE	IDENTIFIER
**Biological samples**		

Human nasal airway epithelial cells (HNEC) from nasal brushing	UMC Utrecht	Protocol ID: 16/586, NL54885.041.16

**Chemicals, peptides, and recombinant proteins**		

Phosphate buffered saline 0, without Ca and Mg (PBS0)	Sigma-Aldrich/Thermo Fisher Scientific/sterile homemade	Cat#D5652; Cat#14190250
Acetic acid	Sigma-Aldrich	Cat#A6283-1L
Collagen IV	Sigma-Aldrich	Cat#C7521
PureCol Type I Collagen Solution	Advanced BioMatrix	Cat#5005
TrypLE express enzyme	Thermo Fisher Scientific	Cat#12605010
Sputolysin	Calbiochem	Cat#560000-10
CryoStor CS10	STEMCELL Technologies	Cat#07930
Advanced DMEM/F-12 (Ad-DF)	Thermo Fisher Scientific	Cat#12634-028
Bronchial epithelial cell medium-basal (BEpiCM-b)	ScienCell	Cat#3211
B-27 Supplement, serum free	Thermo Fisher Scientific	Cat#17504001
GlutaMAX Supplement	Thermo Fisher Scientific	Cat#35050-061
HEPES	Thermo Fisher Scientific	Cat#15630080
3,3′,5-Triiodo-L-thyronine sodium salt	Sigma-Aldrich	Cat#T6397
(±)-Epinephrine hydrochloride	Sigma-Aldrich	Cat#E4642
Hydrocortisone	Sigma-Aldrich	Cat#H0888
N-Acetyl-L-cysteine	Sigma-Aldrich	Cat#A9165
Nicotinamide	Sigma-Aldrich	Cat#N0636
A83-01 (TGF-βi)	Tocris	Cat#2939/10
DAPT (NOTCHi)	Thermo Fisher Scientific	Cat#15467109
DMH-1 (BMPi)	Selleck Chemicals	Cat#S7146
SB 202190 (p38i)	Sigma-Aldrich	Cat#S7067
TTNPB (Retinoic acid agonist)	Cayman	Cat#16144-1
Y-27632 (ROCKi)	Selleck Chemicals	Cat#S1049
Recombinant human fibroblast growth factor 7 (FGF-7)	PeproTech	Cat#100-19
Recombinant human fibroblast growth factor 10 (FGF-10)	PeproTech	Cat#100-26
Recombinant human pro-epidermal growth factor (EGF)	PeproTech	Cat#AF-100-15
Recombinant human hepatocyte growth factor (HGF)	PeproTech	Cat#100-39H
Recombinant neuregulin-1β	PeproTech	Cat#100-03
Recombinant interleukin-1β	PeproTech	Cat#200-01B
RSPO3-Fc fusion protein conditioned medium (R-spondin 3)	U-Protein Express	Cat#R001 - 100 mL
Amphotericin B	Thermo Fisher Scientific	Cat#15290018
Gentamicin	Sigma-Aldrich	Cat#G1397
Penicillin–streptomycin	Thermo Fisher Scientific	Cat#15070-063
Primocin	InvivoGen	Cat#ant-pm-2
Vancomycin	Sigma-Aldrich	Cat#SBR00001
Collagenase type II	Thermo Fisher Scientific	Cat#17101-015
Cultrex Basement Membrane Extract, Type 2, Pathclear (BME)	Trevigen	Cat#3532-010-02
Matrigel® Growth Factor Reduced (GFR) Basement Membrane Matrix, LDEV-free	Corning	Cat#354230
Cell recovery solution	Corning	Cat#354253
VX-445	MedChemExpress	Cat#HY-111772
VX-661	Selleck Chemicals	Cat#S7059
VX-809	Selleck Chemicals	Cat#S1565
VX-770	Selleck Chemicals	Cat#S1144
Forskolin	Sigma-Aldrich	Cat#F3917-10mg
Calcein green acetoxymethyl (AM)	Invitrogen	Cat#C34852

**Software and algorithms**		

Zen Blue Software	Zeiss	https://www.zeiss.com/microscopy/int/products/microscope-software/zen.html
Prism 8	GraphPad Software Inc.	https://www.graphpad.com/scientific-software/prism/
Microsoft Excel	Microsoft Corporation	https://office.microsoft.com/excel
R (version 4.2.2)	R Core Team (2022)	https://www.R-project.org/
RStudio (version 2022.7.2.576)	RStudio Team (2022)	http://www.rstudio.com/
ImageJ/FIJI	Wayne Rasband, NIH, USA	https://imagej.net/Fiji/Downloads

**Other**		

Cytological brush	CooperSurgical	Cat#C0004
Interdental brush 3–5 mm	E.g., Lactona	N/A
pluriStrainer® 30 μm	ITK Diagnostics	Cat#43-50030
pluriStrainer® 100 μm	ITK Diagnostics	Cat#43-57100-51
MACS SmartStrainers (100 μm)	Miltenyi Biotec bv	Cat#130-098-463
12-mm Transwell with 0.4-μm Pore Polyester Membrane Insert	Corning	Cat#3460
6.5-mm Transwell with 0.4-μm Pore Polyester Membrane Insert	Corning	Cat#3470
CELLSTAR® 6-well plate, TC	Greiner Bio-One	Cat#657160
CELLSTAR® 12-well plate, TC	Greiner Bio-One	Cat#655180
CELLSTAR® 24-well plate, suspension	Greiner Bio-One	Cat#662102
CELLSTAR® 96-well plate, F-bottom, μClear®, black, TC	Greiner Bio-One	Cat#655090
CELLSTAR® 96-well plate, U-bottom, TC	Greiner Bio-One	Cat#650180
CoolCell™ LX Cell Freezing Container	Corning	Cat#CLS432001-1EA
Zeiss LSM800 confocal microscope	Zeiss	N/A
Liquid nitrogen tank	N/A	N/A

## Materials and equipment

### Stock preparation of medium compounds

**Timing: 16–20 h**

**Table:** 

Compound	Stock preparation	Stock concentration	Storage
Advanced DMEM/F-12 (Ad-DF)	Ready to use	–	4°C for 1 year
Bronchial epithelial cell medium-basal (BEpiCM-b)	Ready to use	–	4°C for 1 year
B-27 Supplement, serum free	Ready to use	–	−20°C for 1 year
GlutaMAX Supplement	Ready to use	–	4°C for 2 years
HEPES	Ready to use	1 M	4°C for 2 years
3,3′,5-Triiodo-L-thyronine sodium salt	Dissolve 100 mg in 100 mL NaOH (0.2 M) and filter sterilize (0.22 μm)	1 mg/mL	−80°C for 1 year
(±)-Epinephrine hydrochloride	Dissolve 25 mg in 5 mL PBS0 and filter sterilize (0.22 μm)	5 mg/mL	−20°C for 1 year
Hydrocortisone	Dissolve 5 mg in 1 mL Ethanol 100% and subsequently in 49 mL Ad-DF. Filter sterilize (0.22 μm)	100 μg/mL	−80°C for 1 year
N-Acetyl-L-cysteine	Dissolve 3.26 g in 40 mL PBS0 MQ and filter sterilize (0.22 μm)	500 mM	−20°C for 3 months
Nicotinamide	Dissolve 5 g in 41 mL PBS0 MQ and filter sterilize (0.22 μm)	1 M	−20°C for 3 months
A83-01 (TGF-βi)	Dissolve 10 mg in 4.74 mL DMSO	5 mM	−80°C or −20°C for 3 months
DAPT (NOTCHi)	Dissolve 10 mg in 1.16 mL DMSO	20 mM	−20°C for 1 year
DMH-1 (BMPi)	Dissolve 10 mg in 5.2571 mL DMSO	5 mM	−20°C for 2 years
SB 202190 (p38i)	Dissolve 5 mg in 500 μL DMSO	30 mM	−20°C for 2 years
TTNPB (Retinoic acid agonist)	Dissolve 1 mg in 2.9 mL DMSO	1 mM	−20°C for 1 year
Y-27632 (ROCKi)	Dissolve 50 mg in 1.56 mL DMSO	100 mM	−20°C for 2 years
Recombinant human Pro-epidermal growth factor (EGF)	Dissolve 100 μg in 2 mL PBS0-BSA 0.1%	50 μg/mL	−20°C for 1 year
Recombinant human Fibroblast growth factor 10 (FGF-10)	Dissolve 1 mg in 1 mL PBS0-BSA 0.1%	1 mg/mL	−20°C for 1 year
Recombinant human Fibroblast growth factor 7 (FGF-7)	Dissolve 100 μg in 1 mL PBS0-BSA 0.1%	100 μg/mL	−20°C for 1 year
Recombinant human Hepatocyte growth factor (HGF)	Dissolve 100 μg in 0.4 mL PBS0-BSA 0.1%	250 μg/mL	−20°C for 1 year
Recombinant Neuregulin-1β	Dissolve 100 μg in 1333 μL PBS-BSA 0.1 %	10 μM	−20°C for 1 year
Recombinant Interleukin-1β	Dissolve 10 μg in 1000 μL PBS-BSA 0.1 %	10 μg/mL	20°C for 1 year
RSPO3-Fc Fusion Protein conditioned medium (R-spondin 3)	Ready to use	–	−80°C or −20°C for 3 months
Collagen IV	Dissolve 10 mg in filter sterilized (0.22 μm filter) 10 mL acetic acid (0.5 M)	20×	−20°C for 1 year
Collagenase type II	Dissolve 1 g in 50 mL PBS0 MQ	20 mg/mL	−20°C for 1 year
PureCol Type I Collagen Solution	Ready to use	3 mg/mL	4°C for 6 months
Amphotericin B	Ready to use	250 mg/mL	−20°C for 1 year
Gentamicin	Ready to use	50 mg/mL	4°C for 2 years
Penicillin-Streptomycin	Ready to use	–	−20°C for 1 year
Primocin	Ready to use	50 mg/mL	−20°C for 1 year
Vancomycin	Ready to use	100 mg/mL	−20°C for 2 years

### Overview of media

**Table:** Overview of all different media used in the protocol

Name	Abbreviation	Preparation	Application
Collection medium	N/A	Section “preparation of collection medium”	Collection and storage of nasal brushings
Basic basal cell medium	BC medium	Section “basic BC medium”	Basic medium which is used to prepare BC isolation or BC expansion medium
Basal cell isolation medium	BC isolation medium	Section “BC isolation medium”	Basic BC medium with supplements to culture basal progenitor cells the first week after isolation
Basal cell expansion medium	BC expansion medium	Section “BC expansion medium”	Basic BC medium with supplements to expand basal progenitor cells
Freezing medium	Freezing medium	Add 5 μL Y-27632 (stock 100 mM) to 100 mL CryoStor CS10	Medium to freeze basal progenitor cells
Basic ALI-differentiation medium	ALI-diff medium	Section “ALI-diff medium”	Basic medium for ALI-differentiation of basal progenitor cells on Transwell inserts
ALI-differentiation medium + A83-01	ALI-diff phase 1 medium	Section “ALI-diff phase 1 medium”	Medium used for the submerged culturing phase on Transwell inserts
ALI-differentiation medium + A83-01 + neuregulin-1β	ALI-diff phase 2 medium	Section “ALI-diff phase 2 medium”	Medium used the first days after air-exposure on Transwell inserts
ALI-differentiation medium + neuregulin-1β	ALI-diff phase 3 medium	Section “ALI-diff phase 3 medium”	Medium used for the rest of differentiation on Transwell inserts
Airway organoid medium	AO medium	Section “AO medium”	Basic medium for airway organoids
Airway organoid culture medium	AO culture medium	Section “AO culture medium”	Medium for organoid formation and maintenance
Airway organoid FIS assay medium	AO FIS assay medium	Section “AO FIS assay medium”	Medium used to enhance CFTR expression in organoids used in the FIS assay

### Preparation of collection medium

Collection medium is used for the collection and preservation of nasal brushings. It can be stored at 4°C for a maximum of one year.

**Table:** Collection medium

Reagent	Final concentration	Amount
Advanced DMEM/F-12 (Ad-DF)	97.8% (v/v)	489 mL
GlutaMAX Supplement (100×)	1% (v/v)	5 mL
Penicillin-Streptomycin (100×)	1% (v/v)	5 mL
Primocin (50 mg/mL)	100 μg/mL	1 mL
**Total**	**N/A**	**500 mL**

### Preparation of basic BC medium, BC isolation and BC expansion medium

Basic basal cell (BC) medium is first prepared and can be used to make BC isolation medium or BC expansion medium by addition of fresh supplements. Basic BC medium can be stored at −20°C for a maximum of 6 months, if desirable as 10 mL or 40 mL aliquots. BC isolation and expansion medium can be stored at 4°C for 2 weeks. BC isolation medium is used for the culturing of basal progenitor cells the first 7 days after isolation. BC expansion medium is used for further expansion of basal progenitor cells.

**Table:** Basic BC medium

Reagent	Final concentration	Amount
Advanced DMEM/F-12 (Ad-DF)	44% (v/v)	220 mL
Bronchial epithelial cell medium-basal (BEpiCM-b) (100%)	49.6% (v/v)	248 mL
B-27 Supplement, serum free (100%)	2% (v/v)	10 mL
GlutaMAX Supplement (100×)	1% (v/v)	5 mL
HEPES (1 M)	10 mM	5 mL
3,3′,5-Triiodo-L-thyronine sodium salt (1 mg/mL)	100 nM	34 μL
(±)-Epinephrine hydrochloride (5 mg/mL)	0.5 μg/mL	50 μL
Hydrocortisone (100 μg/mL)	0.5 μg/mL	2.5 mL
N-Acetyl-L-cysteine (500 mM)	1.25 mM	1.25 mL
Nicotinamide (1M)	5 mM	2.5 mL
A83-01 (TGF-βi) (5 mM)	1 μM	100 μL
DMH-1 (BMPi) (5 mM)	1 μM	100 μL
SB 202190 (p38i) (30 mM)	500 mM	8 μL
Y-27632 (ROCKi) (100 mM)	5 μM	25 μL
Penicillin-Streptomycin (100×)	1% (v/v)	5 mL
Primocin (50 mg/mL)	100 μg/mL	1 mL
**Total**	**N/A**	**500 mL**

**Table:** BC isolation medium

Reagent	Final concentration	Amount
Basic BC medium (100%)	100% (v/v)	10 mL
Recombinant human Pro-epidermal growth factor (EGF) (50 μg/mL)	5 ng/mL	1 μL
Recombinant human Fibroblast growth factor 7 (FGF-7) (100 μg/mL)	25 ng/mL	2.5 μL
Recombinant human Fibroblast growth factor 10 (FGF-10) (1 mg/mL)	100 ng/mL	1 μL
Recombinant human Hepatocyte growth factor (HGF) (250 μg/mL)	25 ng/mL	1 μL
RSPO3-Fc Fusion Protein conditioned medium (100%)	2% (v/v)	200 μL
Amphotericin B (250 mg/mL)	250 μg/mL	10 μL
Gentamicin (50 mg/mL)	50 μg/mL	10 μL
Vancomycin (100 mg/mL)	50 μg/mL	5 μL
**Total**	**N/A**	**10 mL**

**Table:** BC expansion medium

Reagent	Final concentration	Amount
Basic BC medium (100%)	98% (v/v)	490 mL
DAPT (NOTCHi) (20 mM)	5 μg/mL	125 μL
Recombinant human Pro-epidermal growth factor (EGF) (50 μg/mL)	5 ng/mL	50 μL
Recombinant human Fibroblast growth factor 7 (FGF-7) (100 μg/mL)	25 ng/mL	125 μL
Recombinant human Fibroblast growth factor 10 (FGF-10) (1 mg/mL)	100 ng/mL	50 μL
Recombinant human Hepatocyte growth factor (HGF) (250 μg/mL)	25 ng/mL	50 μL
RSPO3-Fc Fusion Protein conditioned medium (100%)	2% (v/v)	10 mL
**Total**	**N/A**	**500 mL**

### Preparation of ALI-differentiation medium

ALI differentiation (ALI-diff) medium is used for the differentiation of basal progenitor cells and can be stored at 4°C for 2 weeks.

**Table:** ALI-diff medium

Reagent	Final concentration	Amount
Advanced DMEM/F-12 (Ad-DF)	98.4% (v/v)	492 mL
3,3′,5-Triiodo-L-thyronine sodium salt (1.5 mM)	100 nM	34 μL
Hydrocortisone (100 μg/mL)	0.5 μg/mL	2.5 mL
(±)-Epinephrine hydrochloride (5 mg/mL)	0.5 μg/mL	50 μL
A83-01 (TGF-βi) (5 mM)	50 nM	5 μL
TTNPB (Retinoic acid agonist) (1 mM)	100 nM	50 μL
Recombinant human EGF (50 μg/mL)	0.5 ng/mL	5 μL
Penicillin-Streptomycin (100×)	1% (v/v)	5 mL
**Total**	**N/A**	**500 mL**

The following variations of ALI-diff medium are used for different stages of differentiation.

#### ALI-diff phase 1 medium

• Add 4 μL A83-01 (final concentration 500 nM) to 40 mL basic ALI-diff medium.

#### ALI-diff phase 2 medium

• Add 4 μL A83-01 (final concentration 500 nM) and 2 μL Neuregulin-1β (final concentration 0.5 nM) to 40 mL basic ALI-diff medium.

#### ALI-diff phase 3 medium

• Add 2 μL Neuregulin-1 β (final concentration 0.5 nM) to 40 mL basic ALI-diff medium.

### Preparation of airway organoid medium

Airway organoid (AO) medium is the basic medium for airway organoids. It can be stored at −20°C for 6 months or at 4°C for 2 weeks.

**Table:** AO medium

Reagent	Final concentration	Amount
Advanced DMEM/F-12 (Ad-DF)	94.8% (v/v)	474 mL
B-27 Supplement, serum free (100%)	2% (v/v)	10 mL
GlutaMAX Supplement (100×)	1% (v/v)	5 mL
HEPES (1 M)	10 mM	5 mL
N-Acetyl-L-cysteine (500 mM)	1.25 mM	1.25 mL
Nicotinamide (1 M)	5 mM	2.5 mL
A83-01 (TGF-βi) (5 mM)	500 nM	50 μL
Penicillin-Streptomycin (100×)	0.5% (v/v)	2.5 mL
**Total**	**N/A**	**500 mL**

The following variations of AO medium are used.

#### AO culture medium

• Add 10 μL DAPT (final concentration 5 μM), 2 μL FGF-7 (final concentration 5 ng/mL) and 0.4 μL FGF-10 (final concentration 10 ng/mL) to 40 mL AO medium.

#### AO FIS assay medium

• Add 2 μL Neuregulin-1β (final concentration 0.5 nM) and 40 μL Interleukin-1β (stock 10 ng/mL) to 40 mL AO medium.

## Step-by-step method details

### Workflow to culture nasal brushing-derived basal progenitor cells

**Timing: 1–2 months**

**Figure 1:**
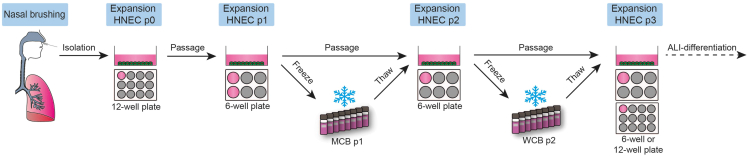
Workflow to culture nasal brushing-derived basal progenitor cells

This part describes the workflow to culture nasal epithelial basal progenitor cells and includes the isolation, expansion and cryostorage of nasal brushing-derived basal progenitor cells (Figure 1).

1. Obtain nasal cells by nasal brushing, according to the section “obtaining nasal cells by nasal brushing”.
2. Expand human nasal epithelial cells (HNEC) passage 0 (p0) in one well of a 12-well plate until a 90% confluent cell layer exists.
3. Passage HNEC p0 cells to 2 wells of a 6-well plate according to the section “passaging and expansion of nasal epithelial basal progenitor cells” for further expansion as HNEC p1 cells.
4. When confluent, freeze HNEC p1 cells as a master cell bank (MCB) according to the section “freezing nasal epithelial basal progenitor cells”.

***Note:*** All cells can be frozen as a MCB or 0.5 × 10^6^ cells can be passaged to another collagen-coated well on a 6-well plate for further expansion.

5. Expand HNEC p2 cells.

***Note:*** Use cells from the previous step or thaw a MCB vial according to the section “thawing and further expansion of cryostored nasal epithelial basal progenitor cells”.

6. Once confluent, freeze as a p2 working cell bank (WCB).

***Note:*** All cells can be frozen as a WCB or 0.5 × 10^6^ cells can be passaged to another collagen-coated well on a 6-well plate for further expansion.

7. Expand HNEC p3 cells.

***Note:*** Use cells from the previous step or thaw a WCB vial according to the section “thawing and further expansion of cryostored nasal epithelial basal progenitor cells”.

8. Once confluent, these cells are ready for differentiation as ALI-cultures.

### Obtaining nasal cells by nasal brushing

**Timing: 15 min**

This section describes the collection of nasal cells through brushing of the inferior nasal turbinates.

#### Preparations

9. Prepare a 15 mL tube with 7 mL collection medium.
10. Prepare a 15 mL tube with 5 mL PBS0.

**Figure 2:**
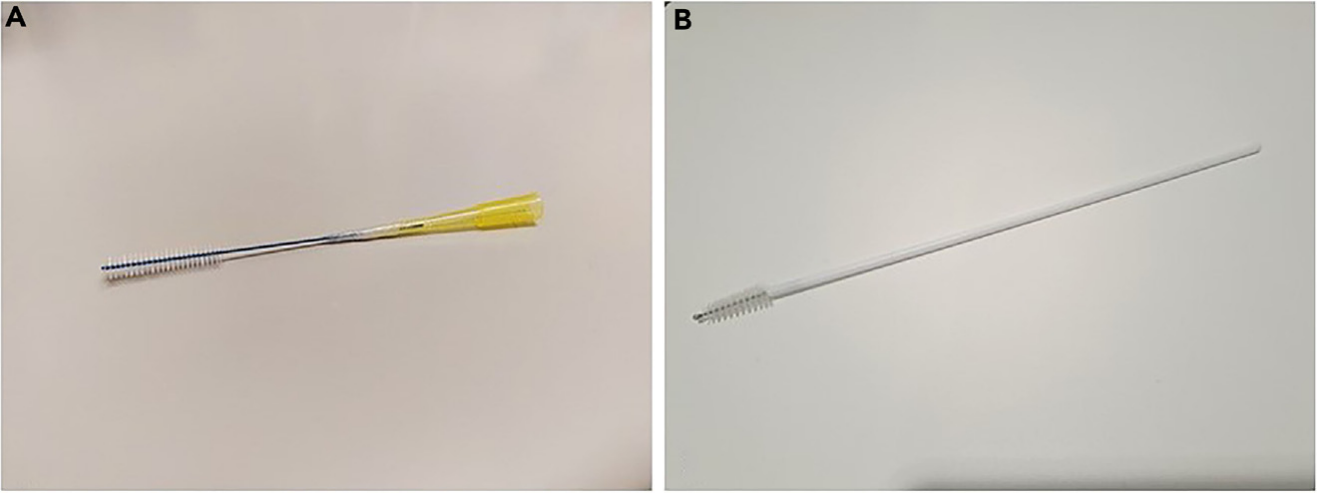
Nasal brushes (A) Nasal brush for younger children, created from an interdental brush connected to a p200 non-filter tip. (B) Cytological brush used for adults.

***Note:*** Smaller nasal brushes may be needed for younger children. These can be created by connecting a p200 non-filter tip to an interdental brush with parafilm (Figure 2).

#### Nasal brushing

11. Inform the nasal cell donor about the procedure and intended use of the cells. Explain that the lacrimal gland will be stimulated by the brushing procedure, which might cause tearing eyes. Infrequently, it causes a nose bleed.
12. Make sure informed consent is documented through signing of appropriate informed consent forms.
13. Ask the donor to blow their nose.
14. Ask the donor to sit straight and sideways to the person taking the brush.
15. Wet the nasal brush in the tube with PBS0.
16. Hold one hand at the back of the head to prevent head movements during the procedure.
17. Insert the brush horizontally into the nostril and the inferior turbinate until mild resistance. Turn the brush twice and remove the brush from the nose.
18. Place the brush in the 15 mL tube with collection medium, properly labeled, and keep on ice.
19. Repeat the procedure for the other nostril with a new brush and place in the same 15 mL tube with collection medium.
20. Cut the top of the brushes and close the 15 mL tube.
21. Maintain the collection tube with brushes on ice or in a refrigerator at 4°C until further processing, which should be performed within 6 h.

### Isolation of nasal epithelial basal progenitor cells from nasal brushes

**Timing: 45–60 min**

This part describes the isolation of nasal epithelial basal progenitor cells from nasal brushes. Half of the cells will be plated for expansion and the other half will be cryopreserved.

**CRITICAL:** All laboratory procedures with nasal cells should be performed in a laminar flow and sterile compounds should be used to prevent contamination.

#### Preparations

22. Coat a well in 12-well plate according to the section “preparation: precoating of well plates for basal progenitor cell expansion”.
23. Prepare and pre-warm BC isolation medium in a water bath at 37°C.
24. Prepare freezing medium, according to the section “overview of media”.

### Isolation of epithelial cells from nasal brushes

**Figure 3:**
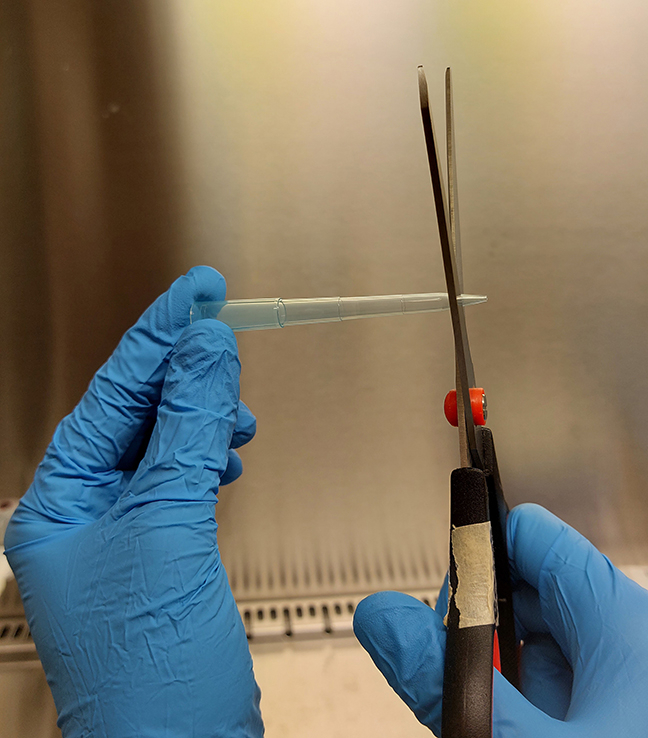
The tip of a non-filter p1000 pipette tip is cut off to make the opening large enough for passing of a nasal brush

***Note:*** This procedure can be performed in a sterile environment with ethanol-cleaned scissors. Another option is to autoclave tips after cutting.

25. Cut the tip (approximately 0.5 cm) of a non-filter p1000 pipette tip with scissors to allow the nasal brush to fit through the opening of the tip (Figure 3).

26. Place one of the brushes temporarily in an empty 15 mL tube to provide space for scraping cells from the brush.
27. Dissociate nasal cells in the tube with collection medium by scraping the brush through the opening of the p1000 pipette tip. Move the brush approximately ten times up and down through the p1000 pipette tip (Figure 4). Discard the brush afterwards.

**Figure 4:**
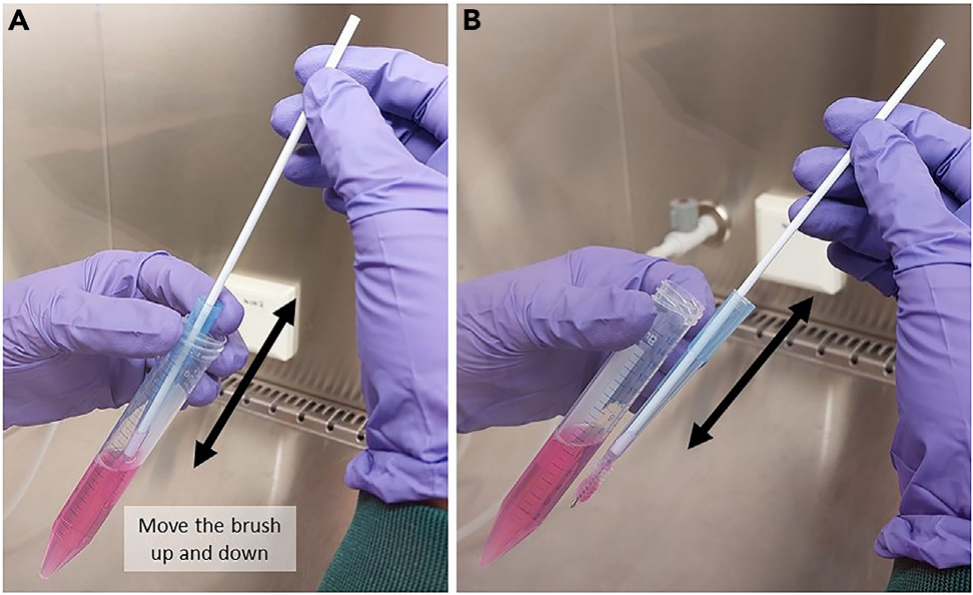
Scraping cells off the brush (A) The brush is moved up and down through the pipette tip to scrape cells from the brush and collect them in the tube with collection medium. (B) A close-up of how to move the brush up and down through the pipette tip. The procedure must be carried out in the collection medium inside the 15 mL tube.
28. Repeat the previous step for the second brush. The cut tip used for the first brush can be re-used.
29. Centrifuge the 15 mL tube with nasal cells at 400 g for 5 min at 4°C.
30. Aspirate the supernatant and resuspend the pellet in 4 mL TrypLE express enzyme + 20 μL Sputolysin (0.5% (v/v)).

**CRITICAL:** Be careful with aspiration when a lot of mucus is present in the pellet to prevent unintended loss of cells.

31. Incubate the cell suspension for 5 min at 37°C (water bath or incubator).
32. Resuspend the cells by pipetting up and down approximately 5 times with a p1000 pipette to dissolve mucus.
33. Incubate the suspension for an additional 5 min at 37°C (water bath or incubator).
34. Add 8 mL of Ad-DF to inactivate the TrypLE express enzyme by dilution.
35. Strain the cell suspension through a 100 μm cell strainer.
36. Wash the strainer with 2 mL Ad-DF.
37. Transfer half of the cell suspension to a new 15 mL tube.
38. Centrifuge both 15 mL tubes at 400 *g* for 5 min at 4°C.
39. Use one 15 mL tube with cells for freezing:
  a. Aspirate the supernatant and resuspend the cell pellet in 500 μL CryoStor CS10 freezing medium (supplemented with 5 μM Y-27632).
  b. Transfer the cell suspension to a sterile cryovial, labeled with donor number.
  c. Gradually freeze the cells by placing the cryovials in a cell freezing container at −80°C.
  d. Transfer the vials to a liquid nitrogen freezer after 24 h.

**CRITICAL:** Cells are first placed in a -80°C freezer for gradual cooling. For optimal cell viability after thawing, cells should be transferred to the liquid nitrogen freezer as soon as possible after reaching a temperature of −80°C.

**Figure 5:**
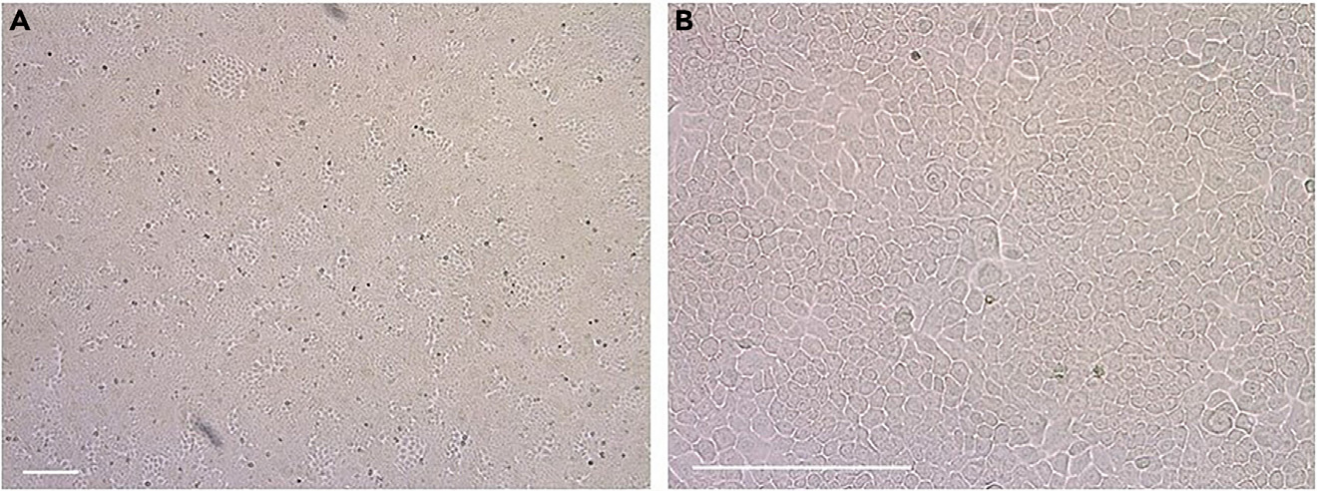
90% confluent nasal epithelial basal progenitor cell culture, ready for passaging (A and B) The cell layer is shown in a low (A) and high (B) magnification. Scale bar equals 200 μm.

38. Use the other 15 mL tube with cells for the expansion of nasal epithelial basal progenitor cells:
  a. Aspirate the supernatant and resuspend the cell pellet in 1 mL BC isolation medium.
  b. Remove the collagen coating solution or PBS0 from the precoated well in a 12-well plate.
  c. Transfer the cell suspension to the precoated well.
  d. Label the plate, indicating passage number (p0) and donor number.
  e. Refresh the medium three times a week, with a maximum of 2 consecutive non-refreshing days, with 1 mL BC isolation medium.
  f. After 7 days, change BC isolation medium for BC expansion medium.
  g. Culture the cells until ∼90% confluence (Figure 5). This will usually take 7–14 days.

**CRITICAL:** It is recommended to culture only one donor per plate because of the risk for infections in HNEC p0 cells.

### Passaging and expansion of nasal epithelial basal progenitor cells

**Timing: 45–60 min**

This part describes the procedure of passaging and further expansion of basal progenitor cells in 12- or 6-well plates. This step is applied as indicated in the section “workflow to culture nasal brushing-derived basal progenitor cells”.

#### Preparations

39. Coat wells in 12- or 6-well plates according to the section “preparation: precoating of well plates for basal progenitor cell expansion”.
40. Prepare and pre-warm BC expansion medium in a water bath at 37°C.

#### Passaging procedure

**Figure 6:**
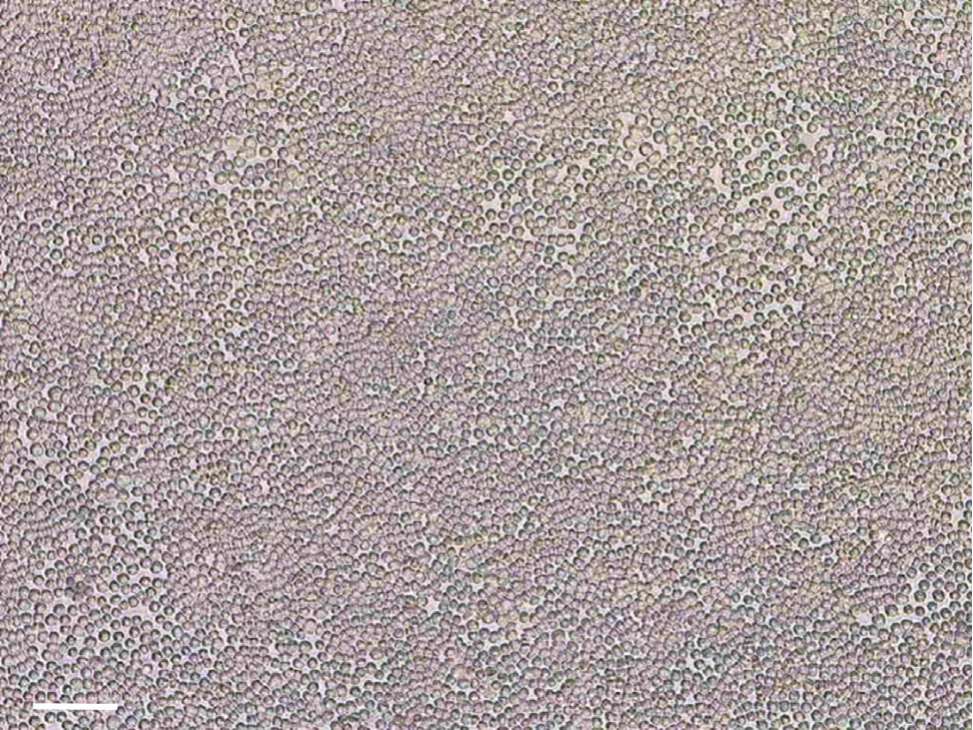
Detached cells after incubation with TrypLE express enzyme Scale bar equals 200 μm.
45. Pipette the TrypLE a few times up and down to detach all cells from the well and transfer the cell suspension to a 15 mL tube.
46. Wash the well with Ad-DF to remove all remaining cells and transfer to the 15 mL tube to inactivate the TrypLE.
  a. For one well in a 12-well plate: 1000 μL Ad-DF.
  b. For one well in a 6-well plate: 2000 μL Ad-DF.
47. Determine cell numbers with a cell counter (e.g., with a BioRad Automated Cell Counter).

***Note:*** The average yield for a confluent well is 1–3 × 10^6^ cells in a 12-well plate and 4–7 × 10^6^ cells in a 6-well plate.

41. Proceed with passaging when the cell culture is approximately 90% confluent (Figure 5).
42. Remove medium and wash the cells with PBS0.
  a. For one well in a 12-well plate: add 500 μL PBS0.
  b. For one well in a 6-well plate: add 1000 μL PBS0.
43. After removal of PBS0, add TrypLE express enzyme to the cells.
  a. For one well in a 12-well plate: add 500 μL TrypLE express enzyme.
  b. For one well in a 6-well plate: add 1000 μL TrypLE express enzyme.
44. Incubate the cells with TrypLE at 37°C until the cells detach. This takes approximately 10–15 min. Check the detachment of the cells under a light microscope (Figure 6). If not detached, incubate for an additional 5 min.

48. Centrifuge the cell suspension at 400 g for 5 min at 4°C.
49. Remove the collagen solution or PBS0 from the precoated wells and transfer the cells dissolved in BC expansion medium to these wells:
  a. For one well in a 12-well plate: add 0.2 × 10^6^ cells to 1 mL BC expansion medium.
  b. For one well in a 6-well plate: add 0.5 × 10^6^ cells to 2 mL BC expansion medium.
50. Refresh the medium three times a week with BC expansion medium, until approximately 90% confluence (Figure 5).
  a. For one well in a 12-well plate: refresh with 1 mL BC expansion medium
  b. For one well in a 6-well plate: refresh with 2 mL BC expansion medium

### Freezing nasal epithelial basal progenitor cells

**Timing: 45–60 min**

This part describes the general procedure of freezing nasal brushing-derived basal progenitor cells after expansion.

#### Preparations

51. Prepare freezing medium, according to the section “overview of media”.

#### Freezing procedure

52. After expansion, detach basal progenitor cells from the well plate according to step 41–46 from the section “passaging and expansion of nasal epithelial basal progenitor cells”.
53. Determine cell concentration with a cell counter (e.g., BioRad Automated Cell Counter).
54. Centrifugate the cell suspension at 400 *g* for 5 min at 4°C.
55. After centrifugation, aspirate the supernatant and resuspend the cells in CryoStor CS10 freezing medium (containing Y-27632) to obtain a cell suspension with a final concentration of 1–2 × 10^6^ cells per mL.
56. Divide 0.5 mL of the cell suspension over sterile cryovials. Depending on the dilution at step 55, each cryovial should contain 0.5–1 × 10^6^ cells. Label the cyrovials, indicating passage number and donor number.
57. Gradually freeze the cells by placing the cryovials in a cell freezing container at −80°C and subsequently transfer them to a liquid nitrogen freezer.

**CRITICAL:** Cells are first placed in a −80°C freezer for gradual cooling. For optimal cell viability after thawing, cells should be transferred to the liquid nitrogen freezer as soon as possible after reaching a temperature of −80°C.

### Thawing and further expansion of cryostored nasal epithelial basal progenitor cells

**Timing: 15–30 min**

This part describes the general procedure of thawing and further expansion of cryostored nasal brushing-derived basal progenitor cells.

#### Preparation

58. Coat wells in 12- or 6-well plates according to the section “preparation: precoating of well plates for basal progenitor cell expansion”.
59. Prepare and pre-warm BC expansion medium.
60. Prepare a 15 mL tube containing 5 mL Ad-DF.

#### Thawing procedure

61. Take a cryovial with cells from the liquid nitrogen freezer and store on dry ice. If not processed immediately, store in the −80°C freezer for a maximum of 14 days.
62. Place the cryovial with cells in a 37°C water bath until the cell suspension is almost thawed and rapidly transfer the cell suspension to the 15 mL tube.
63. Wash the cryovial with 1 mL of Ad-DF and add to the 15 mL tube.
64. Centrifuge at 400 *g* for 5 min at 4°C.
65. Remove the collagen solution or PBS0 from the precoated wells and transfer the cells dissolved in BC expansion medium to these wells:
  a. For one well in a 12-well plate: add 1 mL BC expansion medium.
  b. For one well in a 6-well plate: add 2 mL BC expansion medium.
66. Refresh cell cultures three times a week with BC expansion medium.
  a. For one well in a 12-well plate: refresh with 1 mL BC expansion medium.
  b. For one well in a 6-well plate: refresh with 2 mL BC expansion medium.

### 2D ALI differentiation of nasal epithelial cells

**Timing: 3–6 weeks**

This section describes the 2D ALI differentiation of HNEC. Basal progenitor cells are transferred to Transwell inserts and cultured in submerged conditions with BC expansion medium to obtain a confluent cell layer. Medium is then changed to ALI-diff medium supplemented with the TGF-β inhibitor A83-01 for habituation to a new medium but without directly starting differentiation. In the next step, the apical medium is removed to culture the cells air-exposed to stimulate differentiation towards a pseudostratified airway epithelium, including secretory and ciliated cells. The day of air-exposure is called t = 0. Neuregulin-1β is added to the medium to boost CFTR expression (Table 1).

**Table 1:** Different stages of ALI differentiation

Step	1	2	3	4
Number of days	± 4–7 days	± 4–10 days	3–5 days	13–15 days
Goal	Confluent cell layer	Adaptation to differentiation medium	Start differentiation at air-exposed conditions	Mucociliary differentiation
Medium	BC expansion	ALI-diff phase 1	ALI-diff phase 2	ALI-diff phase 3
				

**Apical compartment**			**Air-exposed**	**Air-exposed**

6.5 mm 24-well insert	200 μL	200 μL	0 μL	0 μL
12 mm 12-well insert	200 μL	200 μL	0 μL	0 μL

**Basal compartment**				

6.5 mm 24-well insert	800 μL	800 μL	600 μL	600 μL
12 mm 12-well insert	1000 μL	1000 μL	1000 μL	1000 μL

A, A83-01; ALI, air-liquid interface; NR, Neuregulin-1β.

#### Preparations

67. Coat Transwell inserts according to the section “preparation: precoating of Transwell inserts for ALI-differentiation”.
68. Prepare and pre-warm ALI-diff medium.

#### Transfer basal progenitor cells to transwell inserts

**Figure 7:**
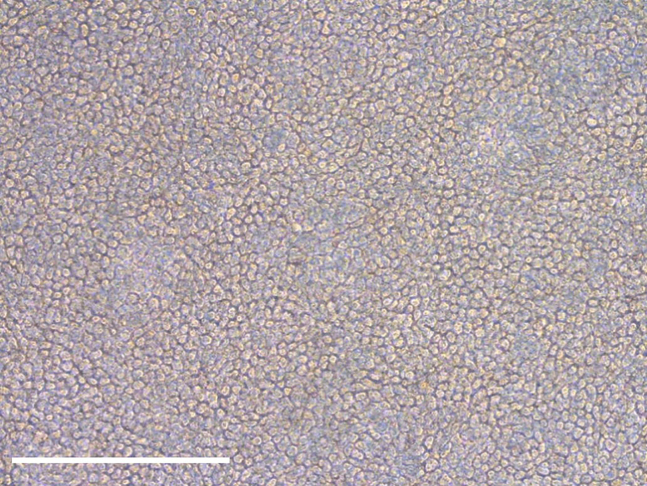
Transwell insert with a 100% confluent cell layer with homogeneous morphology, ready for the switch to air-exposed conditions Scale bar equals 200 μm.
77. Remove medium from the apical side and culture the cells under air-exposed conditions. Change medium at the basolateral side for ALI-diff phase 2 medium (ALI-diff medium supplemented with A83-01 (500 nM) and neuregulin-1β (0.5 nM)).
  a. For 6.5 mm inserts (24-well plate): add 600 μL ALI-diff phase 2 medium.
  b. For 12 mm inserts (12-well plate): add 1 mL ALI-diff phase 2 medium.

***Note:*** The moment of air-exposure is called t = 0 of differentiation.

69. Dissociate basal progenitor cells from a well plate according to step 41–46 from "passaging and expansion of nasal epithelial basal progenitor cells”.
70. Centrifuge the cell suspension at 400 *g* for 5 min at 4°C.
71. Remove the coating solution from the Transwell inserts.
72. Seed basal progenitor cells on the apical side of the Transwell inserts:
  a. For 6.5 mm inserts (24-well plate): 0.2 × 10^6^ cells in 200 μL BC expansion medium.
  b. For 12 mm inserts (12-well plate): 0.5 × 10^6^ cells in 200 μL BC expansion medium.
73. Add BC expansion medium to the basolateral side:
  a. For 6.5 mm inserts (24-well plate): 800 μL BC expansion medium.
  b. For 12 mm inserts (12-well plate): 1 mL BC expansion medium.
74. Refresh the medium three times a week and culture the cells under submerged conditions until full confluence is reached. This takes approximately 4–7 days.
75. When confluent, change apical and basolateral medium for ALI-diff phase 1 medium (ALI-diff medium supplemented with A83-01 (500 nM)).
76. Refresh the medium three times a week until a homogeneous monolayer exists with all cells having a similar size and morphology (Figure 7). This takes approximately 4–10 days.

78. After 3–5 days, replace ALI-diff phase 2 medium for ALI-diff phase 3 medium (ALI-diff medium supplemented with neuregulin-1β (0.5 nM)). Refresh the basolateral medium twice a week.
79. Wash the cells once a week at the apical surface, by incubation with PBS0 in a tissue incubator at 37°C for 5–10 min.
  a. For 6.5 mm inserts (24-well plate): wash with 125 μL PBS0.
  b. For 12 mm inserts (12-well plate): wash with 200 μL PBS0.
80. Differentiation starts from the moment of air-exposure (t = 0) and can be observed by the observation of beating cilia under a light microscope. Cells are ready for the next step when beating cilia are observed, or after 18 days of differentiation.

***Note:*** A high abundance of ciliated cells might cause the unwanted formation of inside-out oriented organoids during the conversion of ALI-differentiated nasal epithelial cells into airway organoids. It is therefore recommended to proceed to the next step as soon as beating cilia are visible and not wait too long before proceeding.

### Conversion of ALI-differentiated nasal epithelial cells into airway organoids

**Timing: 2–3 h**

**Figure 8:**
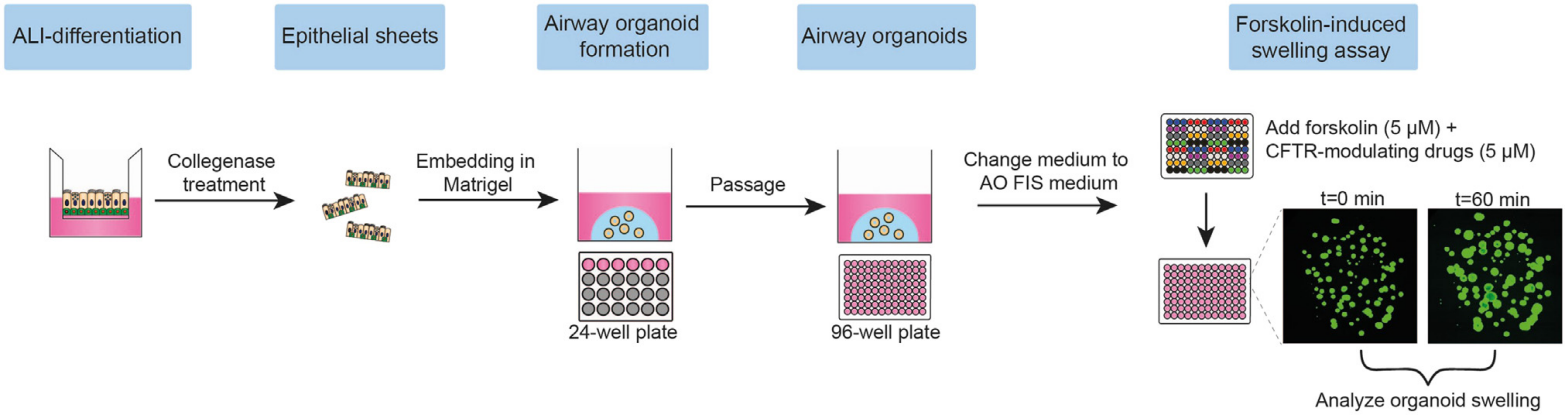
Workflow from ALI-differentiated cells towards airway organoids

This section describes the conversion of ALI-differentiated nasal epithelial cells into airway organoids. The ALI-differentiated epithelial cell layer is detached from the Transwell insert with collagenase. Epithelial sheets are then manually disrupted and strained to generate 30–100 μm fragments to be plated in Matrigel droplets. The complete workflow from ALI-differentiated cell cultures towards airway organoids is shown in Figure 8.

#### Preparations

81. Pre-warm 24-well suspension plates in a tissue incubator for at least 24 h.
82. Thaw Matrigel on ice or at 4°C.
83. Prepare and pre-warm AO culture medium.

***Alternatives:*** BME (100%) can be used as an alternative to Matrigel (100%).

#### Conversion of ALI-differentiated nasal epithelial cells into airway organoids

84. Wash the differentiated ALI-cultures at the apical surface with PBS0. Incubate for 5 min in a tissue incubator.
  a. For 6.5 mm inserts (24-well plate): 125 μL PBS0.
  b. For 12 mm inserts (12-well plate): 200 μL PBS0.
85. Remove the PBS0 or medium at the apical and basolateral side of the ALI-culture. Add collagenase type II solution (1 mg/mL in Ad-DF) to the basolateral side.
  a. For 6.5 mm inserts (24-well plate): 600 μL collagenase solution.
  b. For 12 mm inserts (12-well plate): 1000 μL collagenase solution.
86. Incubate for 45–60 min in a tissue incubator.

***Note:*** The epithelial layer will dissociate from the insert during this procedure.

87. Add Ad-DF to the apical surface of the Transwell insert and transfer the dissociated epithelial layer to a 15 mL tube.
  a. For 6.5 mm inserts (24-well plate): add 100 μL Ad-DF per Transwell insert.
  b. For 12 mm inserts (12-well plate): add 200 μL Ad-DF per Transwell insert.
88. Wash the apical side of the Transwell insert with Ad-DF and transfer remaining epithelial fragments to the 15 mL tube containing the epithelial layer.
  a. For 6.5 mm inserts (24-well plate): 100 μL Ad-DF per Transwell insert.
  b. For 12 mm inserts (12-well plate): 200 μL Ad-DF per Transwell insert.

***Note:*** Epithelial fragments might stick to the edge of the Transwell membrane, which can be checked with a light microscope. In this case, a P200 pipet can be used to scrape the epithelial layer off the membrane.

**Figure 9:**
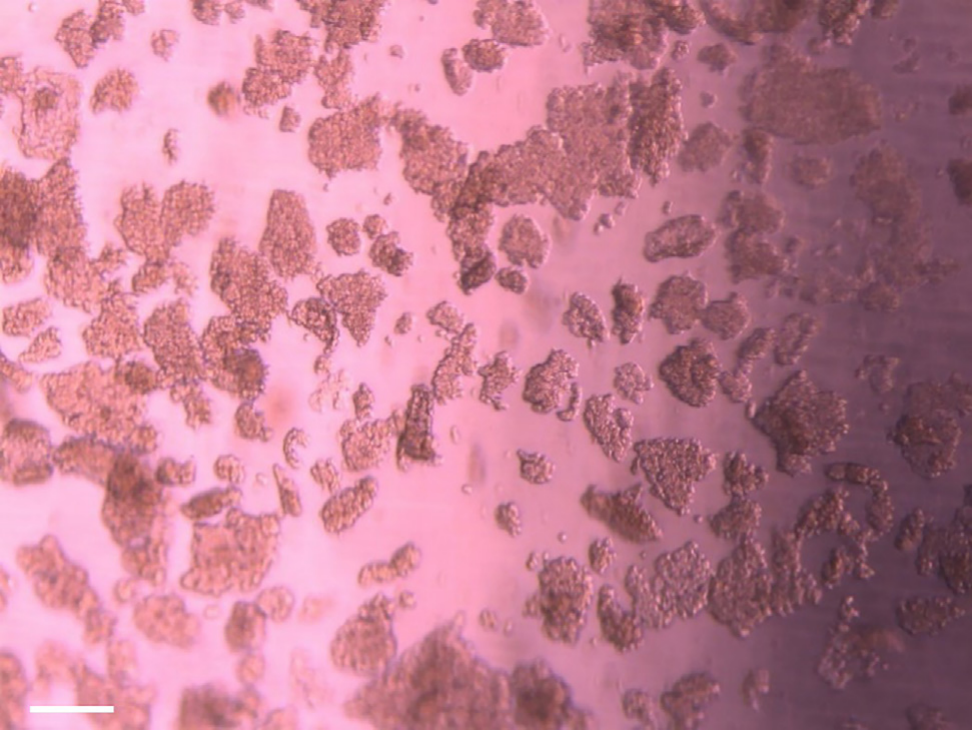
Correct size of epithelial fragments in a 15 mL tube after disruption Scale bare equals 200 μm.

89. Disrupt the epithelial layer into smaller fragments with a P1000 pipet tip. Evaluate the size of the fragments by eye or a light microscope. The majority of the epithelial fragments should have a diameter of approximately 30–100 μm (Figure 9).

***Optional:*** When having difficulties to obtain small fragments, an additional P200 non-filter tip can be placed on top of the P1000 pipet tip.

**Figure 10:**
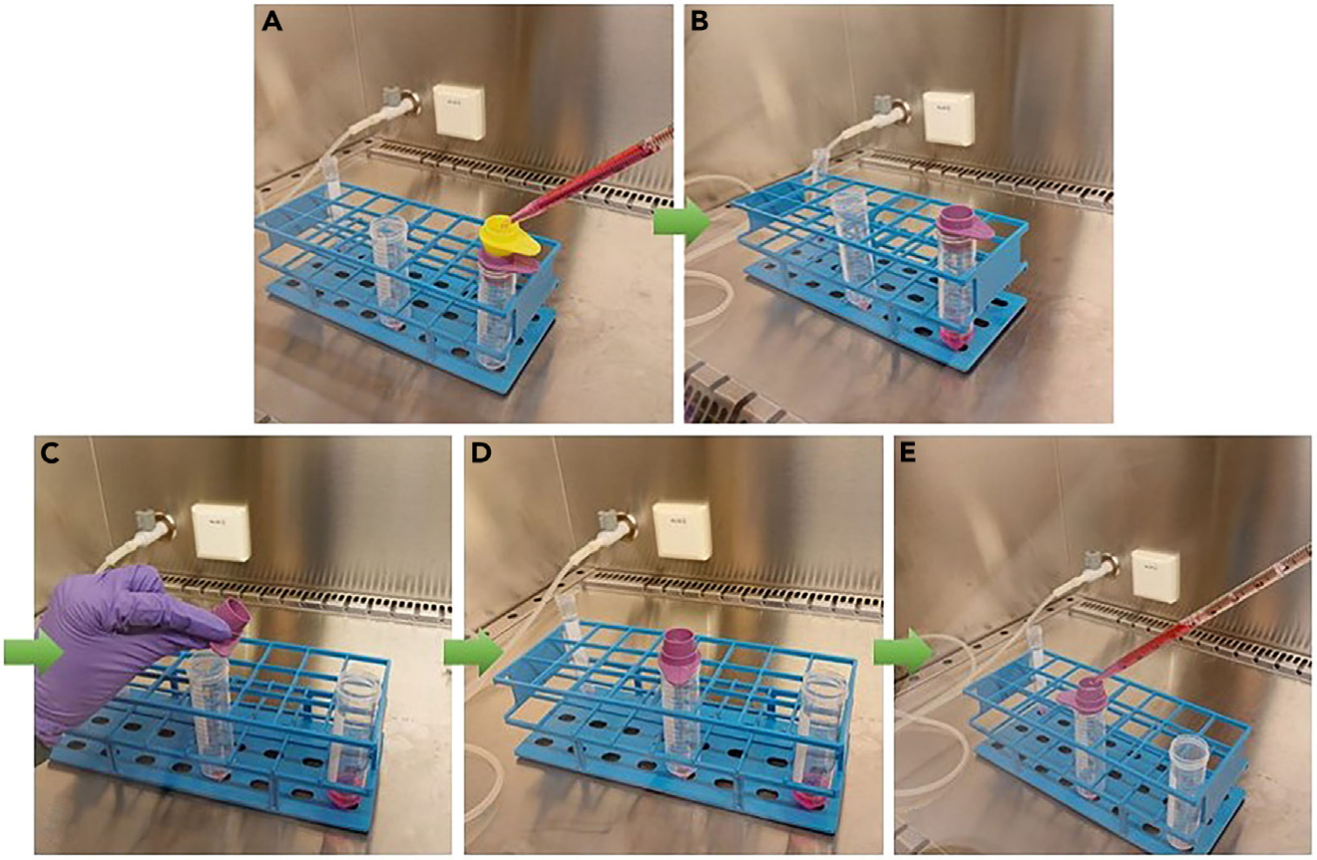
The steps (A–E) of the straining procedure to obtain epithelial fragments with a size between 30-100 μm. A 100 μm (yellow) and 30 μm pluriStrainer® (purple) are used.
  b. Discard the 100 μm strainer (Figure 10B).
  c. Flip the 30 μm strainer upside down on top of a new 50 mL tube (Figures 10C and 10D).
  d. Add 5 mL Ad-DF to the reversed 30 μm pluriStrainer® to collect the epithelial fragments (size between 30–100 μm) in the 50 mL tube (Figure 10E).

***Note:*** Wash both sides of the 100 μm and 30 μm pluriStrainers® before use with 1 mL Ad-DF to enable efficient flow-through.

90. Strain the epithelial fragments with a combination of a 100 μm pluriStrainer® placed on top of a 30 μm pluriStrainer®.
  a. Add an additional 4 mL Ad-DF to the 15 mL tube with epithelial fragments, and transfer the fragments through the strainers (Figure 10A).

91. Transfer the epithelial fragments to a 15 mL tube and centrifuge at 400 *g* at 4°C for 5 min.
92. Remove the supernatant and add 100% Matrigel to the pellet and mix well.
  a. For epithelial fragments from a 6.5 mm 24-well insert: add 60 μL Matrigel, to plate 2 × 30 μL droplets.
  b. For epithelial fragments from a 12 mm 12-well insert: add 180 μL Matrigel, to plate 6 × 30 μL droplets.

**CRITICAL:** This step should be performed on ice to prevent solidification of Matrigel.

**CRITICAL:** Avoid formation of bubbles during resuspension of the epithelial fragments in Matrigel.

**Figure 11:**
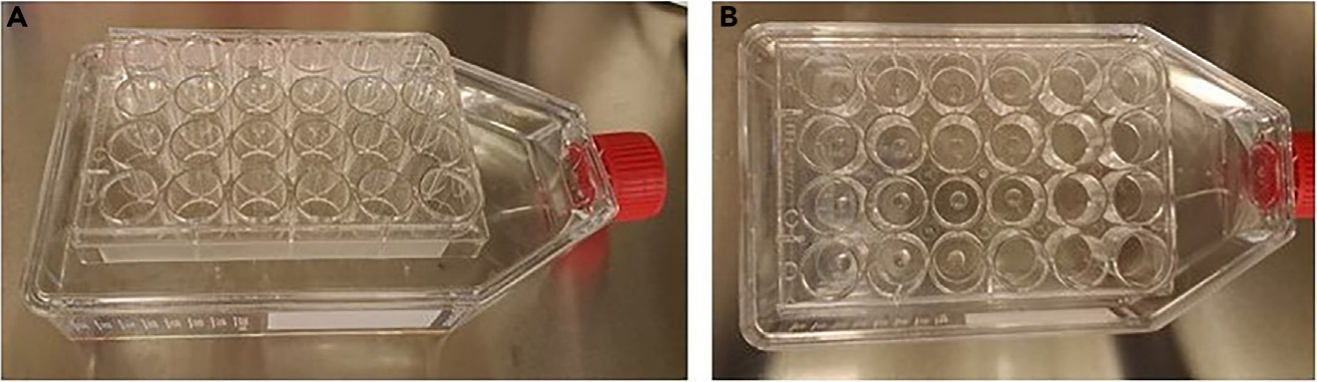
Epithelial fragments embedded in Matrigel are plated in a 24-well plate placed on top of a T175 flask filled with warm water (A) Side view. (B) Top view.

***Note:*** The plate is flipped upside down to distribute the epithelial fragments equally through the Matrigel, enabling equal access to nutrients.

**Figure 12:**
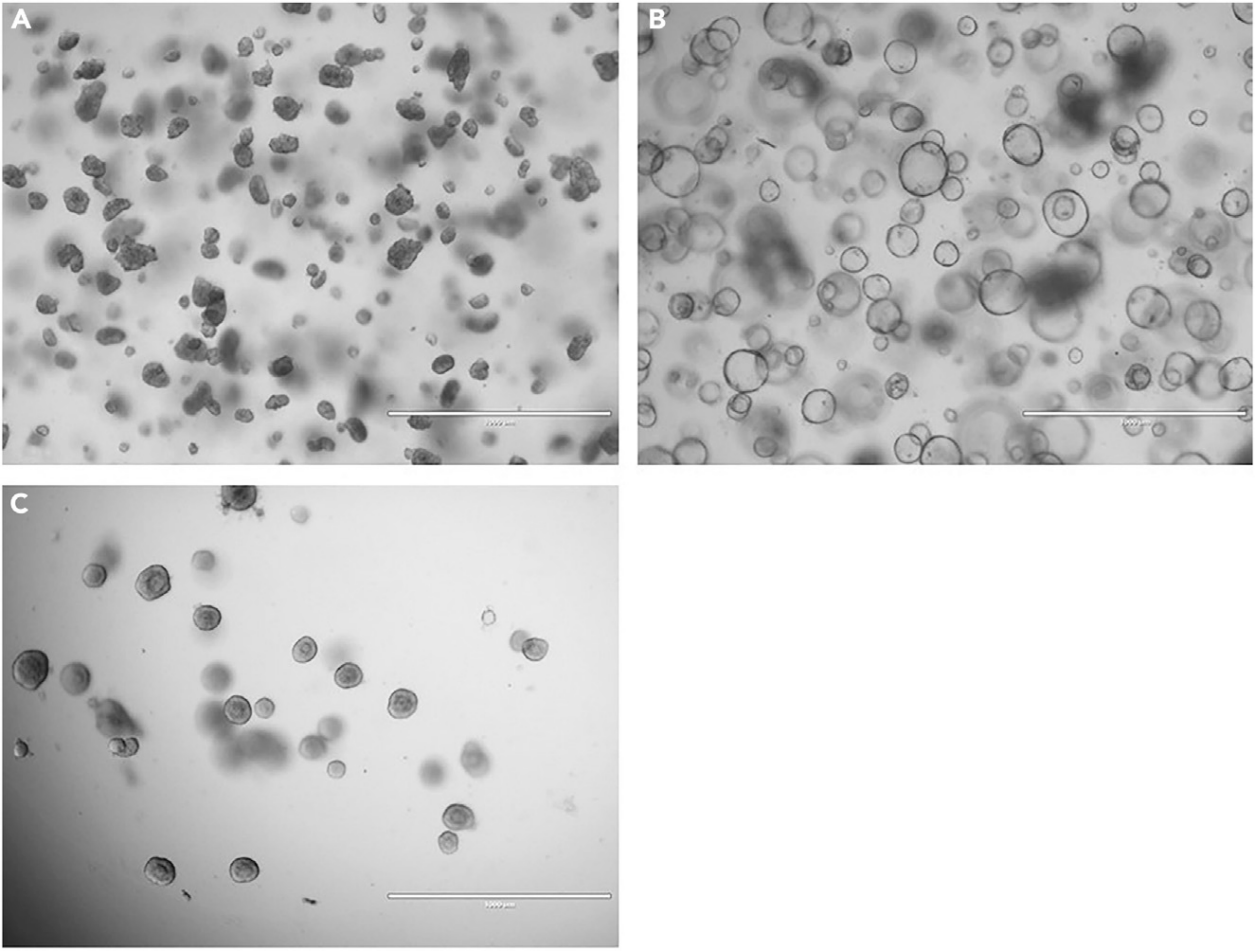
Formation of airway organoids (A) Epithelial fragments embedded in Matrigel, 30 min after plating and solidification of the Matrigel in the 24-well plate. (B) Airway organoids acquired intrinsic lumen, 3 days after plating. (C) Airway organoids lose their intrinsic lumen by changing medium to AO FIS medium, 5 days after medium change and after passaging to a 96-well plate. Scalebar equals 1000 μm.
95. Refresh the medium twice a week.

***Note:*** Gently add the medium to avoid disruption of the Matrigel droplets.

93. Place a pre-warmed 24-well suspension plate on top of a T175 flask filled with handwarm tap water (Figure 11). Apply 30 μL droplets per well. After 2 min, flip the plate upside down and solidify the droplets in a tissue incubator for 15–30 min.

94. Add 500 μL pre-warmed AO culture medium to the epithelial fragments embedded in Matrigel (Figure 12A).

96. Epithelial fragments self-organize into organoids and develop lumen in 3–5 days (Figure 12B). Organoids can be passaged to a 96-well plate after lumen formation.

### Passaging of nasal organoids to a 96-well plate

**Timing: 1–2 h**

This section describes the passaging of nasal organoids to 96-well plates, which can be used for FIS assays.

#### Preparations

97. Pre-warm black flat-bottom tissue culture-treated 96-well plates in a tissue incubator for at least 24 h.
98. Thaw Matrigel on ice or at 4°C.
99. Prepare and pre-warm AO culture medium.

#### Passaging of nasal organoids to 96-well plates

100. Remove culture medium and add 400 μL cell recovery solution per well.
101. Resuspend the Matrigel droplet once in cell recovery solution.
102. Incubate the well plate at 4°C in the fridge for 5–10 min until the Matrigel is dissolved.

***Alternatives:*** Cell recovery solution is recommended to dissolve the Matrigel efficiently. As an alternative, ice cold Ad-DF can be used, in which case the 4°C incubation step can be skipped.

**Figure 13:**
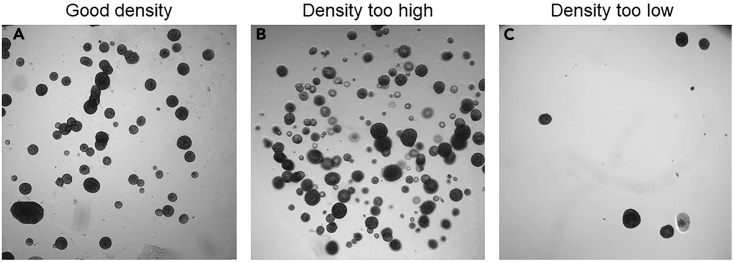
Organoids plated in different densities (A) Well containing an appropriate number of organoids. (B) Well with too many organoids and in different Z planes. (C) Well with too few organoids.

***Note:*** The Matrigel-embedded organoids can be transferred to an Eppendorf tube for easier handling.

103. Transfer the organoids to a 15 mL tube with 7 mL ice cold Ad-DF to inactivate the cell recovery solution. Keep the tube on ice.
104. Centrifuge the organoids at 400 *g* and 4°C for 5 min.
105. Remove supernatant and resuspend the organoid pellet in 100% Matrigel. Keep the tube on ice. The plating volume is 4 μL ∗ amount of wells in 96-well plate with 10% extra volume.
  a. With good quality organoid cultures, one 30 μL Matrigel droplet from a 24-well plate can be seeded into 16 wells of a 96-well plate.
  b. To avoid too sparsely seeded wells, it is recommended to first resuspend the organoids in a Matrigel volume sufficient to seed half of the wells. Plate a 4 μL test droplet and check density under a light microscope. In case the organoid density is too high, organoids can be diluted by adding more Matrigel (Figure 13).

106. Take a pre-warmed 96-well plate and add 4 μL Matrigel droplets with organoids in each well.

**CRITICAL:** Regularly resuspend the organoids in the Eppendorf tube to keep a homogeneous suspension.

**CRITICAL:** Tap the 96-well plate regularly on a flat surface during plating (± after every 16–24 wells) to distribute the organoids in the same plane.

107. Solidify the Matrigel droplets by placing the plate in a tissue incubator for 15–25 min.
108. Add 100 μL AO culture medium to each well and place the plate in a tissue incubator at 37°C and 5% CO2.
109. Refresh the medium twice a week.

### FIS assay for nasal organoids

**Timing: 2–2.5 h**

This section describes the FIS assay which can be used to visualize and quantify the functional rescue of CTFR protein function by CFTR modulator treatment in nasal organoid swelling experiments.

#### Preparations: 5–10 days prior to the FIS assay

110. Replace organoid medium with 100 μL pre-warmed AO FIS assay medium, 5–10 days prior to the FIS assay.

***Note:*** Organoids display complete loss of lumen formation 2–5 days after the media change to AO FIS assay medium (Figure 12C).

#### Preparations: 48 h prior to the FIS assay

111. Refresh the organoid medium with 100 μL AO FIS assay medium supplemented with CFTR correctors (i.e., VX-809, VX-445, VX-661; 5 μM) or vehicle control, 48 h prior to the FIS assay.

#### Preparations: Similar day as the FIS assay

112. Turn on the environmental control of the confocal microscope at least 1 h before start of the experiment at 37°C and 5% CO_2_.
113. Dissolve calcein green (50 μg) in 6 μL DMSO, and prepare calcein green solution (1:750 v/v) in Ad-DF (10 μL/well).
114. Prepare 2× concentrated dilutions of compounds to be added acute: i.e., forskolin (10 μM), CFTR potentiators (VX-770; 10 μM) or vehicle in Ad-DF. Dispense 125 μL of the compound solutions per well in a new U-bottom 96-well suspension plate.

***Note:*** The stimuli will be further diluted 1:1 when added to the well plate with organoids, already containing 100 μL medium. So final concentrations of both forskolin and CFTR potentiators are 5 μM.

#### FIS assay

115. Dispense 10 μL calcein green solution to each well of the 96-well plate with organoids. Mix the calcein green solution through gentle resuspension (2–3 times pipetting) with a multichannel.

***Alternatives:*** Instead of adding the calcein green solution with a multichannel, an automatic repetitive pipet (such as the Eppendorf Multipette E3) with a high dispensing speed could be used.

116. Incubate the plate in a tissue incubator for 30 min.
117. Position the 96-well plate with organoids in the place holder of a confocal microscope and ensure the plate is fixed in position.
118. Set the live cell imaging settings:
  a. Use the 2.5× objective.
  b. Calcein green staining can be visualized with an emission at 488 nm and excitation at 515 nm.
  c. Set the laser intensity, ensuring that the calcein green signal in the organoid structures is slightly oversaturated.
  d. Set the right position (X, Y) and focus (Z) for each well to obtain a good view of the organoids, or use autofocus when possible.
  e. Set a time lapse for 1 h measurement:
    i. Interval = 10 min.
    ii. Cycles = 7 (cycle 1 is t = 0).

***Optional:*** If possible, include brightfield/DIC imaging to obtain a better view of organoid morphology.

119. Add 100 μL from the 96-well plate with acute stimuli to the well plate with organoids, using a multichannel. Immediately start imaging after addition of the acute stimuli.

**CRITICAL:** It is important to start imaging quickly after addition of the acute stimuli, as organoids might directly start swelling.

## Expected outcomes

The FIS assay with intestinal organoids is already an established and widely used assay to predict CFTR modulator efficacy on individual basis.^2^^,^^3^ However, it is proposed that an airway model might be more convenient as people with CF are mostly suffering from respiratory symptoms. Furthermore, a nasal brushing is less invasive compared to an intestinal biopsy. Here we describe a protocol to perform the FIS assay in nasal brushing-derived airway organoids, of which we recently showed their ability to predict CFTR modulator responses.^1^ To guarantee evenly differentiated organoids, we differentiated basal progenitor cells as ALI-cultures before their transformation into organoids.^1^ This will diminish the variation in organoid morphology and in FIS between individual organoids in a single well.

By use of this protocol, investigators should be able to isolate, expand and differentiate basal progenitor cells from nasal brushes, to generate nasal airway organoids and to conduct the FIS assay to determine CFTR function and CFTR modulator response efficacy.

From nasal brushings of one donor, approximately 5 × 10^6^ cells can be isolated and cryostored as a MCB p1 (10 vials containing 5 × 10^5^ cells). Each MCB vial can be further expanded in the next passage (p2) to a yield of approximately 5 × 10^6^ cells, indicating that in total approximately 5 × 10^7^ basal progenitor cells (p2) can be generated from the MCB and 5 × 10^8^ cells at a higher passage (p3). These basal progenitor cells (p3) are transferred to Transwell filters for ALI-differentiation. 5 × 10^5^ basal progenitor cells are seeded on a 12 mm 12-well insert, which yields approximately 48–96 wells with organoids in a 96-well plate. When smaller Transwell inserts are used, 2 × 10^5^ basal progenitor cells are seeded on a 6.5 mm 24-well insert, which yields approximately 16–32 wells with organoids in a 96-well plate.

**Figure 14:**
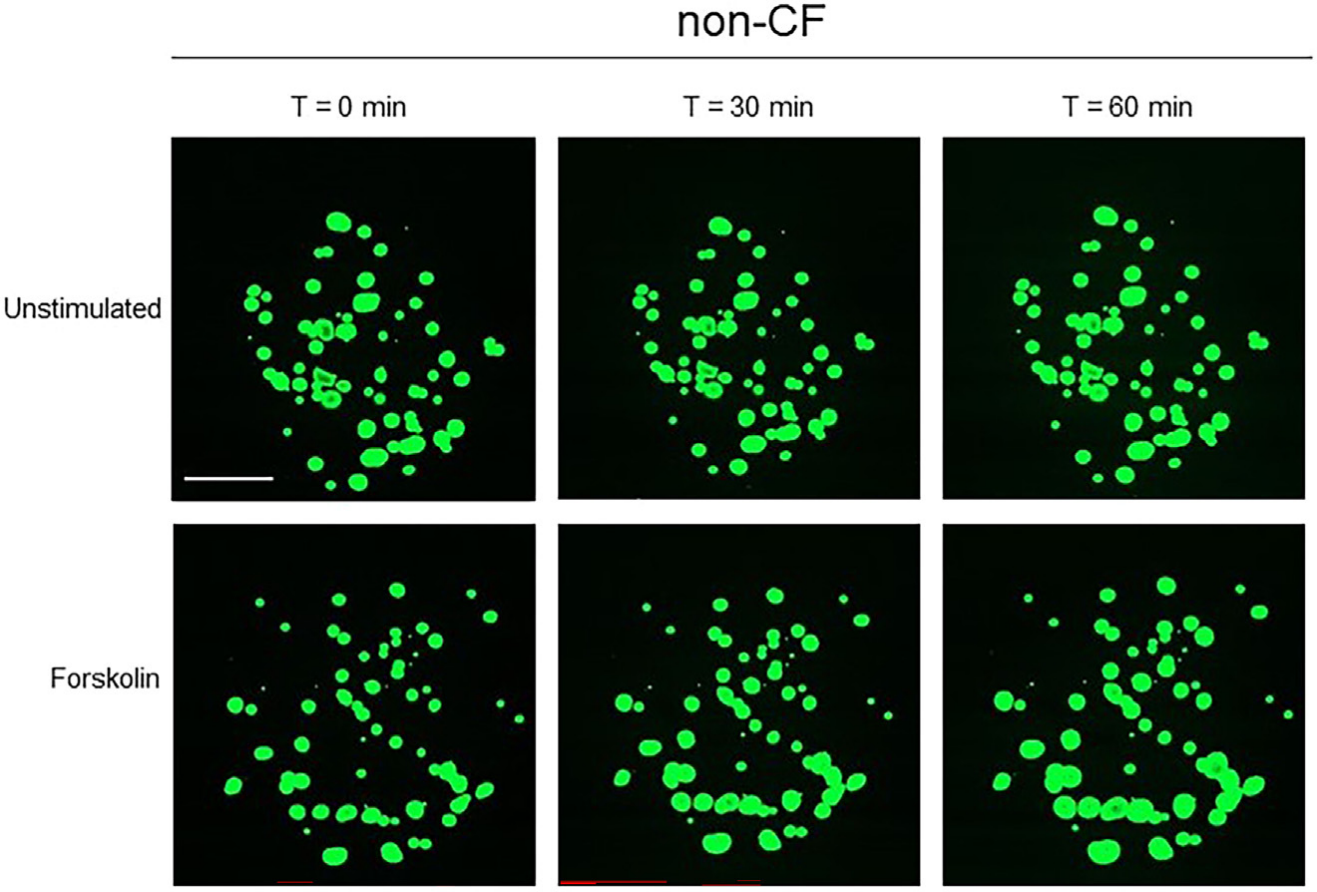
Confocal images of calcein green stained organoids from a non-CF donor, unstimulated or stimulated with forskolin Scale bar equals 500 μm.

**Figure 15:**
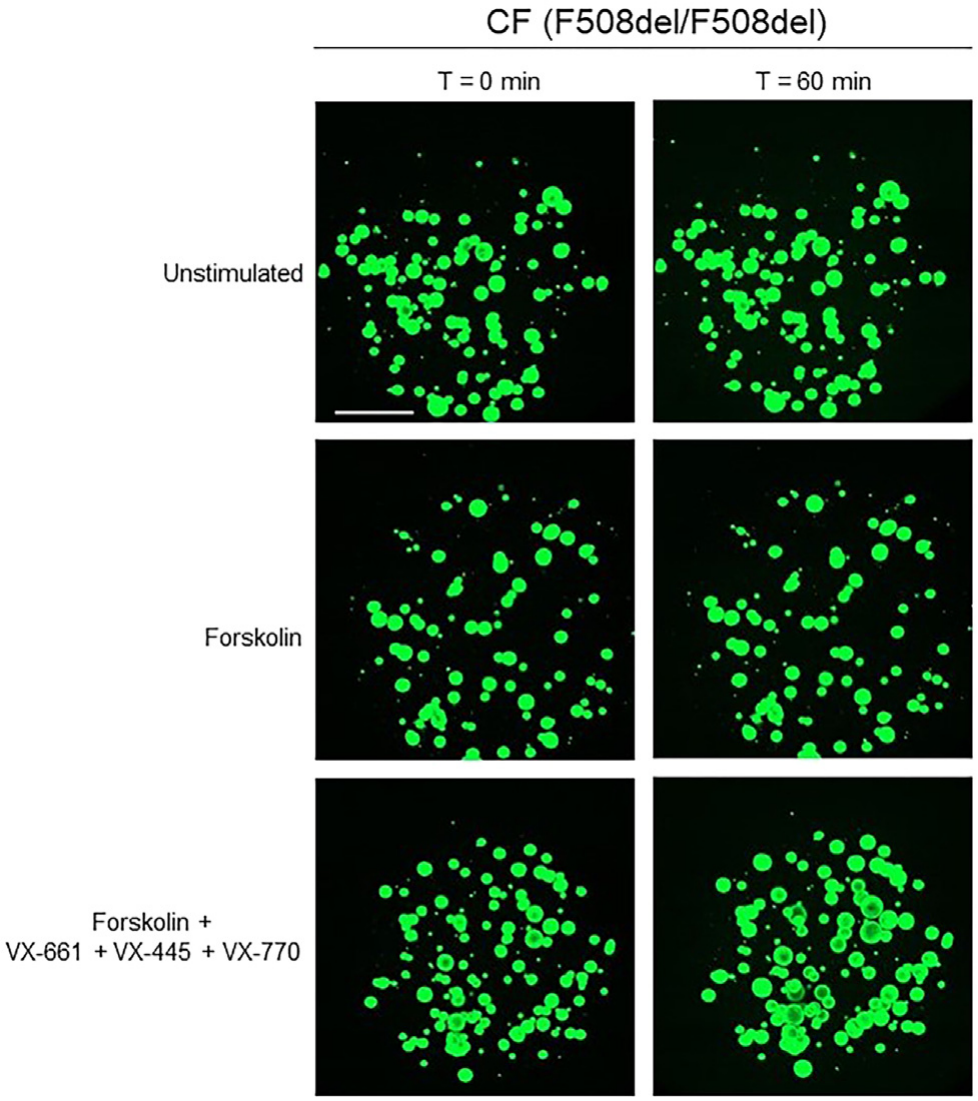
Confocal images of calcein green stained organoids from a CF donor (F508del/F508del) unstimulated or stimulated with either forskolin alone or with forskolin + CFTR modulator treatment (48h pre-incubation with VX-661 and VX-445 and acute stimulation with VX-770) Scale bar equals 500 μm.

Figures 14 and 15 illustrate example results of a FIS assay experiment. In non-CF nasal organoids, forskolin stimulation induces organoid swelling (Figure 14). CF nasal organoids lack swelling in response to forskolin, but the swelling response is restored upon treatment with CFTR modulators (Figure 15).

## Quantification and statistical analysis

The output of the FIS assay is a time series of confocal images containing calcein green stained organoids. For quantification and interpretation of the results, organoid swelling is calculated as the relative increase of total organoid area over time, according to the following steps.

1. Determination of total organoid area in each image (Table 2):
  a. Total organoid area is determined in each individual image using image analysis software, such as Zen blue (Zeiss), or open-source software, such as ImageJ or CellProfiler.^4^ Further processing can be performed with Excel, Graphpad or R.
  b. Particles with a size smaller than 6.000 μm^2^ are excluded from analysis.

**Table 2:** Example dataset showing total organoid area (mm^2^) per well and per time point for a FIS experiment

Time (min)	Well number + condition															
	1	2	3	4	5	6	7	8	9	10	11	12	13	14	15	16
	Unstimulated				Forskolin				Forskolin + Vx-809 + Vx-770				Forskolin + Vx-661 + Vx-445 + Vx-770			
0	1.63	1.98	1.70	1.86	1.97	2.01	1.95	1.74	2.26	1.91	1.96	1.58	1.81	2.07	1.80	2.11
15	1.69	2.01	1.78	1.91	2.09	2.09	2.04	1.80	2.38	2.04	2.09	1.73	2.22	2.49	2.15	2.61
30	1.71	2.02	1.81	1.94	2.19	2.17	2.13	1.86	2.52	2.18	2.26	1.89	2.64	2.93	2.52	3.06
45	1.73	2.04	1.83	1.95	2.23	2.23	2.17	1.89	2.58	2.25	2.34	1.97	2.96	3.27	2.81	3.42
60	1.74	2.04	1.83	1.97	2.26	2.26	2.19	1.90	2.60	2.28	2.37	2.00	3.19	3.54	3.05	3.69

Quadruplicates are used as technical replicates within an experimental plate.

**Figure 16:**
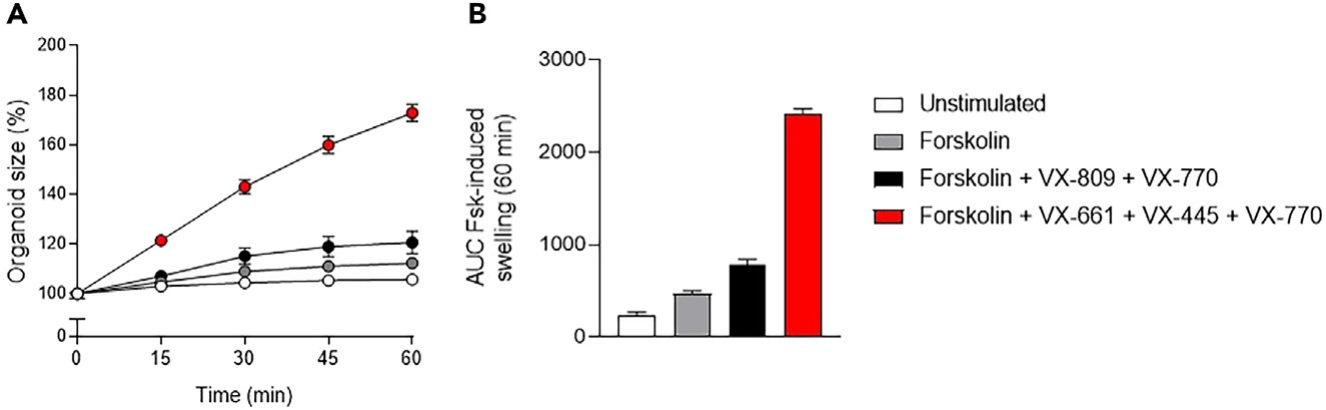
Quantification of a FIS assay with the experimental data from Table 2 and Table 3 Panel A shows normalized organoid area (%) over time and panel B shows the corresponding AUC values. Data is shown as mean ± SD from 4 technical replicates per condition. Fsk = forskolin.

2. Normalization of total organoid area to baseline (Table 3):
  a. Organoid swelling is usually expressed as relative increase to baseline (t = 0), which is set at 100%. The formula to calculate normalized organoid area for individual wells at a specific time point x is:
    $$Normalized\;organoid\;area\;at\;t=x=\frac{Total\;organoid\;surface\;area\;at\;t=x}{Total\;organoid\;surface\;area\;at\;t=0}\times 100\%$$
  b. The average normalized organoid area (%) of technical replicates within a plate can be plotted against time (minutes) to visualize the kinetics of organoid swelling (Figure 16A).
    ***Note:*** Individual wells can be excluded from analysis when less than 10 organoids are present in a well, or when out of focus organoids are not recognized in all time points of the experiment.

**Table 3:** Normalized organoid area (%) of the experimental dataset from Table 2

Time (min)	Well number + condition															
	1	2	3	4	5	6	7	8	9	10	11	12	13	14	15	16
	Unstimulated				Forskolin				Forskolin + Vx-809 + Vx-770				Forskolin + Vx-661 + Vx-445 + Vx-770			
0	100	100	100	100	100	100	100	100	100	100	100	100	100	100	100	100
15	103.6	101.4	104.5	102.4	106.1	104.0	105.1	103.6	105.3	107.0	106.9	109.4	123.0	120.4	119.2	123.2
30	105.0	102.3	106.4	104.0	111.4	108.2	109.6	106.8	111.5	114.2	115.7	119.3	146.1	141.6	139.9	144.6
45	106.2	102.9	107.5	104.6	113.5	110.9	111.4	108.4	114.1	118.0	119.5	124.3	163.7	158.0	156.1	161.8
60	106.6	103.2	107.7	105.4	114.9	112.6	112.6	109.3	115.1	119.7	121.3	126.3	176.7	171.3	169.0	174.5

Total organoid area is normalized to baseline (t = 0, 100%).

3. Calculation of area under the curve (AUC) values as a measure for organoid swelling:
  a. AUC values are calculated as a measure for organoid swelling. This creates a single data point per well which can be used to compare organoid swelling in response to different stimuli.
  b. Mean AUC values of technical replicates within a plate can be visualized with bar graphs (Figure 16B).
4. Statistical analysis:
  a. Statistical analysis can be performed to compare different groups with a student t-test or ANOVA, dependent on the number of groups.

## Limitations

This protocol describes the use of primary nasal cell cultures. It is important to realize that working with primary cell cultures is different compared to cell lines. Primary cell cultures have an increased risk of microbial outgrowth during the first day of cell isolation, especially in cell cultures derived from individuals with CF. Furthermore, donor to donor variation exists, such as differences in the expansion rate of basal progenitor cells, in differentiation time of ALI-cultures and in the organoid yield. This variation might be related to donor characteristics, to the yield of cells from the nasal brushing, to environmental conditions or to the freeze/thawing procedure. It is recommended to prevent changes in medium batches and guarantee correct freeze/thawing procedures. Be aware that changing manufacturer of medium compounds or using alternative cultureware may affect the quality of the cell and organoid cultures.

## Troubleshooting

### Problem 1

Fungal infection of basal cell cultures, related to the section “isolation of nasal epithelial basal progenitor cells from nasal brushes”.

### Potential solution

Add the antifungal reagent Fungin (50 μg/mL) to the cell culture medium, wrap the plate in foil, keep the cell culture isolated from other cell cultures and check if the infection disappears in the coming 3 days. If not, the cell culture should be thrown away to prevent contamination of other cell cultures.

### Problem 2

**Figure 17:**
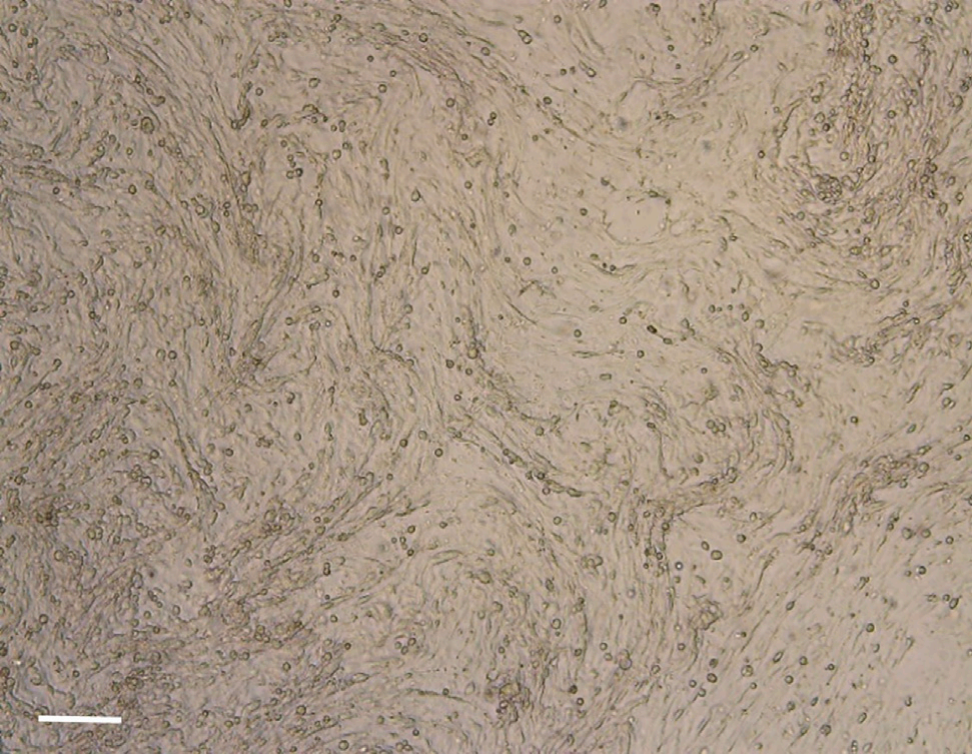
Squamous cell differentiation of basal progenitor cells Scale bar equals 200 μm.

Squamous cell differentiation in basal cell cultures or ALI-cultures related to the section “passaging and expansion of nasal epithelial basal progenitor cells” or “2D ALI differentiation of nasal epithelial cells” (Figure 17).

### Potential solution

In general, basal progenitor cells displaying squamous cell differentiation cannot be used for ALI-differentiation or organoid formation.

When squamous cell differentiation occurs in multiple donors cultured in parallel, it might be caused by incorrect preparation of the culture medium, e.g., the use of compounds past their expiring date, or incorrect storage or preparation of stock compounds or media batches. Renew compounds or medium when needed.

The occurrence of squamous cell differentiation might also relate to a specific donor. Thaw and expand cells of other vials from this donor and determine whether squamous cell differentiation occurs. If this is the case, the cells of this donor cannot be further used. Consider to repeat the isolation of nasal cells from the frozen back up vial or to collect new nasal brushes.

### Problem 3

Cells have difficulty starting up after thawing, related to the section “thawing and further expansion of cryostored nasal epithelial basal progenitor cells”.

### Potential solution

Thaw cells in a smaller wells, or thaw cells from a lower passage number to create a new working cell bank.

### Problem 4

Inside out-oriented organoids, related to the section “conversion of ALI-differentiated nasal epithelial cells into airway organoids”.

### Potential solution

When differentiated ALI cultures display high numbers of ciliated cells, this may impede formation of organoids with the lumen facing inward. Cilia beating in highly ciliated epithelial fragments prevents solidification of the Matrigel, leading to formation of inside out-oriented organoids. Therefore, cells should not be differentiated too long at the ALI and should be converted into organoids as soon as cilia appear during differentiation.

### Problem 5

Nonoptimal organoid density in 96-well plates for a FIS assay experiment, related to the section “passaging of nasal organoids to a 96-well plate”.

### Potential solution

For optimal performance of the FIS assay, it is important to seed organoids in the right density in 96-well plates (Figure 13). A high seeding density might cause problems for organoid recognition during image analysis, especially when organoids are in different planes. A low seeding density will provide unreliable results.

### Problem 6

Organoids swell during the experiment without any stimulation, related to the section “FIS assay for nasal organoids”.

### Potential solution

Check temperature and CO_2_ settings of the microscope incubator as organoid swelling might be pH dependent.

## Resource availability

### Lead contact

Further information and requests for resources and reagents should be directed to and will be fulfilled by the lead contact, Jeffrey Beekman, j.beekman@umcutrecht.nl.

### Materials availability

This study did not generate new unique reagents.

## Data Availability

This study did not generate datasets or code.
